# Alcohol Intake and Incidence of Heart Failure and Its Subtypes: VA Million Veteran Program

**DOI:** 10.3390/nu18030471

**Published:** 2026-01-31

**Authors:** Xuan-Mai T. Nguyen, Eiman Elhouderi, Yanping Li, April R. Williams, Liam Gaziano, Jacob Joseph, John Michael Gaziano, Kelly Cho, Luc Djousse

**Affiliations:** 1 Massachusetts Veterans Epidemiology Research and Information Collaborative, VA Boston Healthcare System, Boston, MA 02111, USAapril.williams9@va.gov (A.R.W.);; 2 Department of Medicine, University of California, Los Angeles, CA 90095, USA; 3 Department of Medicine, Corewell Health, Grand Rapids, MI 49546, USA; 4 Department of Medicine, Brown University, Providence, RI 02912, USA; 5 VA Providence Healthcare System, Providence, RI 02908, USA; 6 Department of Medicine, Harvard Medical School, Boston, MA 02115, USA; 7 Division of Aging, Department of Medicine, Brigham and Women’s Hospital, Boston, MA 02115, USA

**Keywords:** alcohol, heart failure, heart failure with reduced ejection fraction, heart failure with preserved ejection fraction, epidemiology, veterans

## Abstract

Background: Little is known about the relation between total alcohol intake and beverage types with the risk of heart failure (HF) and its subtypes in the veteran population. This study aims to examine the associations between total and type of alcohol consumption and risk of HF and its subtypes, namely HF with reduced [HFrEF] and preserved [HFpEF] ejection fraction, in a large cohort of US veterans. Methods: The study cohort included 401,348 Million Veteran Program participants with complete alcohol information collected through a survey and no HF at baseline. HF events were defined as 1 inpatient or 1 outpatient diagnosis code together with at least two ejection fraction (EF) measurements. We defined HFrEF as HF with left ventricular ejection fraction (LVEF) of ≤40% and HFpEF as heart failure with LVEF ≥ 50%. The associations between alcohol intake, type of beverage consumed (i.e., beer, wine, or liquor), and incidence of HF, HFpEF, and HFrEF were assessed using Cox proportional hazard models. Restricted cubic spline regression was used to assess for a dose–response association between alcohol consumption and the risk of HF. Results: Mean age was 65 years, and 91% were men. With a mean follow-up of 6.4 years, we observed 38,420 incident HF events (15,356 HFrEF, 19,047 HFpEF, and 4017 HF with an EF value of 41–49%). Compared to never drinkers, multivariable adjusted hazard ratios for HF were 0.90 (95% CI: 0.86, 0.94), 0.88 (95% CI: 0.84, 0.93), 0.86 (95% CI: 0.81, 0.91), 0.92 (95% CI: 0.86, 0.98), 0.95 (95% CI: 0.84, 1.06), and 1.08 (95% CI: 1.01, 1.15) for current drinkers of 0.1–0.5, 0.6–1, 1.1–2, 2.1–3, 3.1–4 drinks/day, and heavy drinkers (i.e., >4 drinks/day and/or those diagnosed with alcohol use disorder), respectively. We found a similar association between alcohol intake and risk of HFpEF and HFrEF, except heavy drinking was significantly associated with HFrEF (HR: 1.13, 95% CI: 1.02, 1.24), not HFpEF (HR: 1.05, 95% CI: 0.96, 1.13). Types of alcoholic beverage preference did not influence the alcohol-HF relation. Conclusions: Our data are consistent with a J-shaped relation between alcohol consumption and risk of heart failure, irrespective of subtypes.

## 1. Introduction

Heart failure (HF) affects an estimated 6 million Americans and is a major clinical and public health concern with significant mortality and morbidity [1]. It is often subtyped based on left ventricular ejection fraction (EF) into two categories: reduced EF (HFrEF) and preserved EF (HFpEF) [1]. Multiple etiologies for HF have been identified, including ischemic heart disease, genetic cardiomyopathy, infiltrative cardiac diseases, myocarditis, and substance abuse, such as cocaine and alcohol [2]. Alcohol intake may have several major direct effects on cardiovascular function, including mitochondrial dysfunction, oxidative stress, and cellular apoptosis and myocytolysis that lead to structural changes in the heart ventricles, which are all linked to risk for HF [3,4,5]. Alcohol intake is common, with approximately 32.5% of the global population consuming between 0.7 and 1.7 standard drinks a day on average [6]. Within the United States (US) alone, more than half of the population consumes alcohol [6]. Moreover, the prevalence of heavy drinking (i.e., drinking five or more drinks for men, or four or more drinks for women, on at least one occasion in the past year) is 25% in the US population [7]. Therefore, it is important to understand the association of alcohol with human health, including the risk for HF.

The association between alcohol consumption and HF in large population studies is inconclusive. Some studies have reported a relation between light to moderate alcohol intake and lower risk of HF [8,9,10], while others found no association [11]. Regarding the type of alcoholic beverage consumed, there are limited studies suggesting that wine might confer more beneficial effects on cardiovascular health than other types of alcoholic beverages [12,13]. There is currently no data available on the relationship between alcohol intake and risk of HF and its subtypes, where drinking patterns and beverage type (beer, wine, or liquor) are considered. Thus, the objective of the current exploratory study was to examine the association between alcohol intake and beverage preference and incidence of HF and its subtypes of HFrEF and HFpEF among a large, multi-ethnic cohort of US veterans.

## 2. Materials and Methods

### 2.1. Population

The Veterans Affairs (VA) Million Veteran Program (MVP) is an ongoing national prospective cohort study that began enrollment in 2011 to study genetic and non-genetic influences on veterans’ health. Of the 1,016,584 veterans enrolled in MVP as of September 2024, nearly half (*n* = 475,121) of the participants completed the MVP Lifestyle Survey that collected information about socioeconomic status and lifestyle factors. After excluding those with prevalent HF (*n* = 37,574), implausible death records (*n* = 662), non-records of clinical visits after baseline (*n* = 19,168), and participants who did not complete all of the alcohol intake questions of the Food Frequency Questionnaire (FFQ) on the MVP Lifestyle Survey (*n* = 16,369), the final analytic sample comprised data for 401,348 MVP participants (Figure S1). The excluded population was relatively elderly, with a lower proportion of females and a higher proportion of diabetes, COPD, and stroke (Table S1).

### 2.2. Incident HF and HF Subtypes

The outcomes of interest in this study were incident HF and two subtypes: HFpEF and HFrEF. HF Patients with incident HF were identified as those with an ICD-9 code of 428.x or ICD-10 code of I50.x and an echocardiogram performed within 6 months of diagnosis [14]. HF events were defined as 1 inpatient or 1 outpatient diagnosis code together with at least two ejection fraction (EF) measurements used to describe HFpEF and HFrEF. HF subtype HFrEF was defined as HF with a left ventricular ejection fraction (LVEF) of ≤40%, and HF subtype HFpEF as HF with LVEF ≥ 50%. EF was measured within 6 months of HF diagnosis. A more detailed description of HF assessment in MVP has been previously described [15,16]. Person-time follow-up started on the date of MVP Lifestyle Survey completion and ended with loss to follow-up, defined as either the occurrence of HF, death, the latest clinical visit, or the end of the follow-up period (30 September 2024).

### 2.3. Alcohol Intake and Preference Assessment

Alcohol intake was the independent variable used in this study. The quantity of alcohol intake and preferred type of beverage were assessed using self-reported responses to the FFQ.

Participants who did not report any alcohol intake during the prior years or “never or less than once per month” for all three alcohol categories (i.e., wine, beer, and liquor) or responded “No, I have never drunk alcohol” to the question about whether they drank alcohol were categorized as “Never drinkers.” Those who responded “No, but I used to drink alcohol” were categorized as “Former drinkers.” Those who responded, “Yes, I currently drink alcohol”, were considered current drinkers, and subsequent questions were used to calculate drinks/day for categorization in the intake.

Participants were asked about their average intake per standard serving of drinks, wine (4 oz.), beer (1 glass, bottle, or can), and liquor (1 drink or shot) during the prior year. Pre-specified response categories were “Never or less than once per month,” “1–3/month,” “1/week,” “2–4/week,” “5–6/week,” “1/day,” “2–3/day,” “4–5/day,” and “6+/day,” which were converted to drinks/day. Total alcohol consumption was the sum of the total drinks of wine, beer, and liquor. Alcohol use disorder (AUD) was defined as participants who had an International Classification of Diseases (ICD)-9 diagnostic code of 303.0 or 305.0 or an ICD-10 diagnosis code of F10.10, F10.20, F10.21, or F10.229 in VA electronic health records. Participants were then categorized into “Never drinkers”, “Former drinkers”, and “Current drinkers” with drinks per day for ranges “0.1–0.5”, “0.6–1”, “1.1–2”, “2.1–3”, “3.1–4”, and “AUD and/or heavy drinkers (>4 drinks/day)”.

Ethanol content was assumed to be 14 g per standard serving of drinks [17]. Total ethanol g/d was calculated by multiplying the average alcohol content in each beverage by the reported total number of drinks consumed in a day. Beverage preference (i.e., beer, wine, or liquor) was determined by whether or not a single beverage contributed to >50% of total ethanol consumed; otherwise, participants were classified as having a preference of mixed type. In a sensitivity analysis, we defined a preference based on whether or not a single beverage contributed to >70% of total ethanol consumed.

### 2.4. Covariates

Information on covariates, including age, body mass index (BMI), race and ethnicity, family income, current marital status, education level, smoking, and exercise frequency, was collected through self-reported surveys at baseline. If self-reported information on age, race/ethnicity, sex, smoking, weight, and height was missing from the surveys, information was obtained from VA electronic health records.

Dietary intake was self-reported through FFQ. Participants were asked “how often” they consume a standard portion of each food item in the past year. Frequencies and proportions of each food item were converted to average daily intake for each participant. Sodium intake was calculated by multiplying the frequency of consumption for each food item by its sodium content from the Harvard University Food Composition Database and summing across all foods. The Dietary Approaches to Stop Hypertension (DASH) score [18,19], was derived based on the consumption of fruits, vegetables, nuts and legumes, whole grains, low-fat dairy, sodium, red and processed meats, and sugar-sweetened beverages.

Medical comorbidities, including atrial fibrillation (AF), diabetes, hypertension, hyperlipidemia, cancers, depression, chronic obstructive pulmonary disease (COPD), stroke, myocardial infarction (MI), and statin medication use, were ascertained in previous work [15,16] using data from VA electronic health records (Table S2). These comorbidities were chosen to control for confounding with HF and HF subtype outcomes [20].

### 2.5. Statistical Analysis

We used Cox proportional hazard models to estimate crude and adjusted hazard ratios (HR) of the outcomes of incident HF, HFpEF, and HFrEF, respectively, comparing former drinkers and current drinkers to participants who never drank alcohol. Person-time follow-up started on the date of MVP Lifestyle Survey completion and ended with either the occurrence of HF, death, the latest clinical visit time, or the end of the follow-up period (30 September 2024).

The first models adjusted for age (continuous); race/ethnicity (non-Hispanic white, non-Hispanic Black, Hispanic, and other race/ethnicities); sex (male/female); education level (≤high school or General Educational Development (GED), some college, or college or above); income level (<$30,000, $30,000–$59,000, ≥$60,000 or missing); and marital status (currently married: yes, no, or missing). The second model further adjusted for frequency of vigorous physical activity (never/rarely, 1–4 times/month, 2–4 times/week, or ≥5 times/week); smoking status (current, former, or never smoking); DASH score (quintile); BMI (kg/m^2^: <18.5, 18.5–22.4, 22.5–24.9, 25.0–27.4, 27.5–29.9, 30–32.4, 32.5–34.9, and ≥35); statin use; and baseline comorbidities including AF, diabetes, hypertension, hypercholesterolemia, depression, COPD, stroke, MI, and all cancers. In secondary analyses, we excluded former drinkers (*n* = 131,830) and used Cox proportional hazard models to study the association between HF risk and alcohol beverage preference.

To quantify a linear trend of relative risk (RR) of HF across alcohol consumption categories, we assigned the median value within each group (zero for both never and former drinkers) and modeled this variable continuously. We also tested for potential nonlinearity in the association between alcohol consumption and the risk of HF using restricted cubic spline regression. Three knots (0, 0.07, and 2.57 drinks/day) were applied to flexibly model the association between alcohol consumption and risk of HF, with the minimum value as the reference level. Nonlinearity in the dose–response relationship of alcohol consumption with the risk of HF was evaluated by comparing the model with the linear term to the model with the linear and cubic spline terms using the likelihood ratio test.

Data collected in MVP for use in this study followed a protocol approved by the Department of Veteran Affairs Central IRB (protocol code MVP001, approved in 2010), and consent from participants was obtained in accordance with the guidelines of the Declaration of Helsinki.

Analyses were conducted using data for participants who enrolled in MVP through 31 July 2024, contained in the MVP Roster version 24.1, a curated research-ready dataset containing demographic and health data matched and validated with data derived from the VA Corporate Data Warehouse [21] (CDW) and the Observational Medical Outcomes Partnership (OMOP) [22]. Availability of MVP data and/or samples is governed by the scope of MVP informed consent and VA policies and requires scientific review by appropriate VA review committees. Inquiries can be directed to the MVP Office (mvploi@va.gov; https://www.research.va.gov/MVP/research.cfm, accessed on 28 January 2026). The SAS Enterprise Guide 8.3 (SAS Institute Inc., Cary, NC, USA) was used to conduct all analyses and produce the figures.

## 3. Results

**Figure 1:**
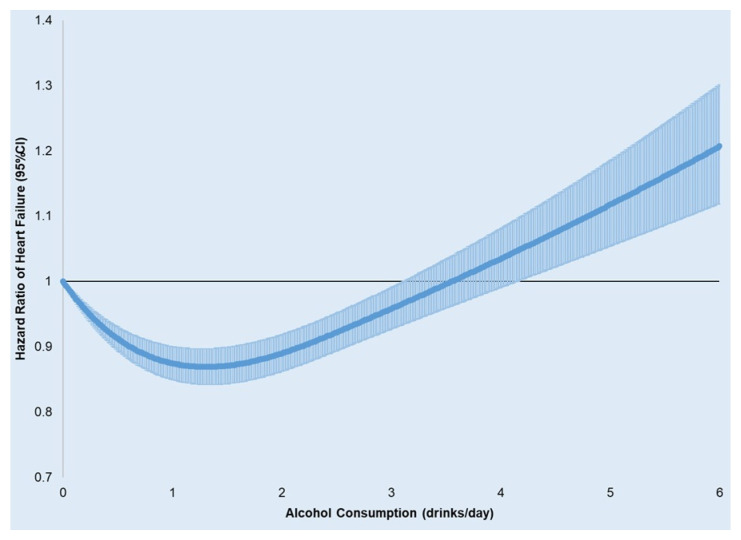
Spline regression of alcohol consumption (drinks/day) and hazard ratios (95% CI) for incident HF. (adjusted for age, gender, race/ethnicity, education level, marital status, income level, exercise frequency, body mass index, DASH score, smoking, statin use, and baseline comorbidities including atrial fibrillation, diabetes, hypercholesterolemia, depression, COPD, stroke, myocardial infarction, and cancers).

Of 401,348 participants, the mean age was 65 years, and 91% were men (Table 1). During a mean follow-up period of 6.4 years, we observed 38,420 incident HF events (15,356 HFrEF, 19,047 HFpEF, and 4017 HF with an EF value of 41–49%). The multivariate-adjusted Cox proportional hazard model showed a J-shaped relation of alcohol with HF, with the lowest risk among participants reporting alcohol consumption of 0.6 to 2 drinks per day. Compared with never drinkers, the hazard ratios (95% CI) were 0.90 (0.86, 0.94), 0.88 (0.84, 0.93), 0.86 (0.81, 0.91), 0.92 (0.86, 0.98), 0.95 (0.84, 1.06) and 1.08 (1.01, 1.15) for subjects consuming alcohol 0.1–0.5, 0.6–1, 1.1–2, 2.1–3, 3.1–4 drinks/day, and AUD and/or heavy drinkers, respectively, after adjustment for age, BMI, race, sex, smoking, education level, income level, exercise frequency, marital status, DASH score, statin use, and baseline comorbidities including AF, diabetes, hypertension, hypercholesterolemia, depression, COPD, stroke, MI and all cancers (Table 2 and Figure 1). The results were consistent in several sensitivity analyses (Table S3) and sub-group populations (Table S4); AUD and/or heavy drinkers were significantly associated with HF among males (HR: 1.08, 95% CI: 1.02, 1.15), ever smokers (HR: 1.13, 95% CI: 1.05, 1.22), and participants without CVD at baseline (HR: 1.09, 95% CI: 1.02, 1.17), Table S4.

**Figure 2:**
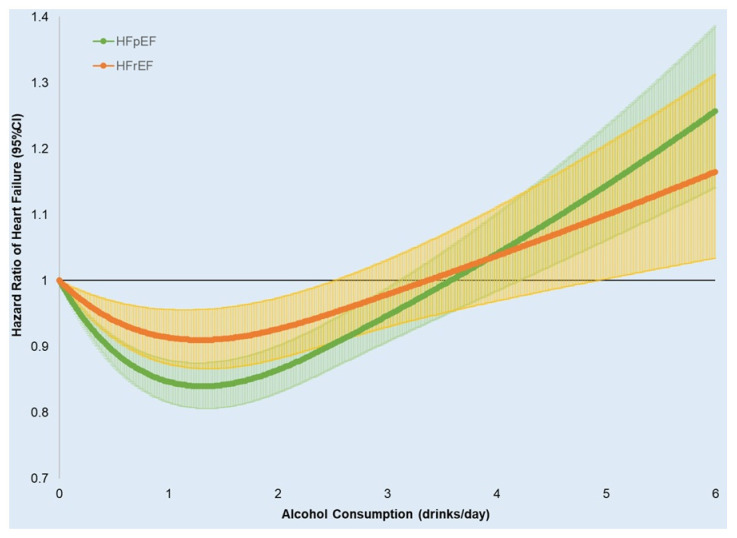
Spline regression of alcohol consumption (drinks/day) and hazard ratios (95% CI) for incident heart failure subtypes. (adjusted for age, gender, race/ethnicity, education level, marital status, income level, exercise frequency, body mass index, DASH score, smoking, statin use, and baseline comorbidities including atrial fibrillation, diabetes, hypercholesterolemia, depression, COPD, stroke, myocardial infarction, and cancers).

A similar J-shaped association was observed for both HFpEF and HFrEF (Table 3 and Table 4 and Figure 2). AUD and/or heavy drinkers are significantly associated with HFrEF (HR: 1.13, 95% CI: 1.02–1.24, Table 3) but not HFpEF (HR: 1.05, 95% CI: 0.96, 1.13, Table 4).

In secondary analyses that evaluated the role of beverage preference on HF incidence, the association with HF was still significant for participants regardless of their preference for beer, wine, liquor, or mixed type (Table 5), even after we defined the preference with a higher cutoff point (Table S5).

## 4. Discussion

We observed that light to moderate alcohol consumption was associated with reduced risk, while heavy drinking was associated with increased risk of HF and its subtypes in the Million Veteran Program. This is consistent with previous studies assessing the association of alcohol intake and HF [8,9,10,23].

### 4.1. Alcohol and Heart Failure Subtypes, HFrEF, and HFpEF

The relationship between alcohol intake and HF has been established with findings from several meta-analyses and large population studies [23,24,25], where higher intake above three drinks per day carries a higher risk for HF, which was identified with findings from the present study. However, existing data on the association between the exact range of alcohol intake and risk of individual HF subtypes (i.e., HFpEF and HFrEF) are limited. One cross-sectional study by Yousaf and colleagues described U-shaped associations for left ventricular dysfunction (defined as LVEF ≤ 40%), with an inverse association for light drinkers (Odds Ratio (OR): 0.14, 95% CI: 0.04, 0.43) and increased odds for heavy drinkers (OR: 4.75, 95% CI: 1.18, 15.98) [26]. Similarly, in our study, we found an increased risk for HFrEF among the AUD and/or heavy drinkers. An analysis of 28,820 individuals from four cohorts, including 982 and 909 incident HFpEF and HFrEF events, respectively, found a lower risk of both HFpEF (HR: 0.74, 95% Cl: 0.59, 0.94) and HFrEF (HR: 0.80, 95% CI: 0.65, 0.98) when comparing those who consumed ≥1 drink per day versus all others [27]. Our findings of an attenuated risk for HF subtypes for lower alcohol consumption may be attributed to our study’s larger sample size and greater number of HF subtype cases, such that we were able to adjust extensively for potential confounding factors and observe more HF events in our population compared with prior published studies. Of interest, the mechanisms by which the different HF subtypes are directly affected by alcohol intake may include oxidative stress, cardiac fibrosis, and excitation-contraction coupling to lower functional EF. Indirectly, the sympathetic nervous system, renin–angiotensin–aldosterone system, and endothelial dysfunction can lead to blood pressure dysregulation [28]. Specifically, compared with those without, those who have structural cardiac aberrations may be classified with a higher risk for HF subtypes when consuming lower ranges of alcohol [29]. These are important to address in future work on alcohol intake and HF subtypes.

### 4.2. Beverage Preference and HF

Research suggests that the protective effects of alcohol on HF might be related to the effect of ethanol on neurohormones that are involved in the progression of HF [30]. It is also possible that some benefits of alcohol on HF may be attributable to the polyphenol content in certain alcoholic beverages, where wine and beer are rich in phenolic content, and liquor is not [31,32,33]. In fact, there are studies suggesting that specific alcoholic beverages, such as red wine, confer a lower risk for heart disease incidence and mortality [34]. However, these studies are often limited as they do not compare risk across different beverage types in their populations. When we examined different alcoholic beverage types and risk for HF, HFrEF, and HFpEF, we observed that the risk of HF and its subtypes did not differ by preference for beer, wine, or liquor. This is consistent with findings from the Kaiser Permanente medical cohort and the Established Populations for the Epidemiologic Study of the Elderly program [30,35]. Additional studies examining the specific content of various phenolic compounds found in different alcoholic beverages and associated risk for HF are warranted.

### 4.3. Study Strengths and Limitations

There are multiple strengths of this study; primarily, the large, multi-ethnic cohort with accompanying linkages to historical health records provided ample power for incident modeling and sensitivity analyses. The large sample size with a high incidence of HF cases allowed for comprehensive adjustment for potential confounding and the ability to examine the differential effects of alcohol on HF subtypes.

This study has limitations that include the potential for different types of bias that should be considered when interpreting the findings. First, this study relies on self-reported alcohol intake, which is subject to misclassification and response bias. Although this study was not intended to establish a causal relationship, we attempted to account for reverse causation by controlling for never and former drinker status. We excluded current drinkers who did not complete the FFQ, which might result in selection bias. Of note, our study population consisted primarily of white men, which can limit the generalizability of the study findings. Lastly, alcohol intake was only assessed at study baseline and does not consider associations with the risk of HF with possible changes in drinking habits over time.

## 5. Conclusions

Our large prospective study showed a J-shaped relationship between alcohol intake and both HFpEF and HFrEF among MVP participants, suggesting a protective effect for HF with light to moderate alcohol intake. Comparing alcoholic beverage types, no specific one offered a protective effect over the others with respect to the risk of both HFpEF and HFrEF. Future work will examine whether the observed associations in this study are further influenced by genetic variations in alcohol metabolism.

## Figures and Tables

**Table 1:** Baseline characteristics of 401,348 participants of the Million Veteran Program by alcohol consumption.

			Current Drinkers (Drinks/Day)						
Characteristic ^1^	Never Drinkers	Former Drinkers	0.1–0.5	0.6–1	1.1–2	2.1–3	3.1–4	AUD ^2^ &/or Heavy Drinkers (>4)	Total
*n*	29,913	131,830	132,030	44,117	22,751	17,549	3804	19,354	401,348
Age, year	67.5 (12.5)	65.9 (11.3)	63.8 (13.1)	65.5 (12.8)	67.2 (12.9)	66.9 (11.1)	67.3 (11.3)	61.7 (11.8)	65.2 (12.4)
Body Mass Index, kg/m^2^	29.4 (5.7)	29.6 (5.8)	29.7 (5.5)	28.7 (4.8)	28.2 (4.6)	28.1 (4.6)	28.1 (4.3)	28.7 (5.2)	29.3 (5.4)
Female, %	13.1	8.4	12.6	6.7	5.2	4.3	2.3	6.2	9.4
Race and ethnicity, %									
Non-Hispanic White	76.1	78.4	78.2	82.8	85.2	87.6	88.7	76.4	79.4
Non-Hispanic Black	12.9	11.7	10.6	8.4	7.2	5.7	5.6	12.5	10.5
Hispanic	6.4	6.6	7.4	5.9	4.9	4.4	3.9	7.6	6.6
Others	4.6	3.4	3.7	2.9	2.7	2.3	1.9	3.5	3.4
Education level, %									
High school	2.8	3.4	1.5	1.3	0.9	1.4	1.0	1.5	2.2
Some college	17.4	20.9	14.2	12.7	11.1	14.8	11.3	17.1	16.5
College or above	64.5	59.9	69.4	73.4	77.1	72.9	78.2	60.0	66.6
Missing	15.3	15.8	14.9	12.5	11.0	10.9	9.5	21.4	14.8
Family annual income, %									
<30 k	25.8	29.2	20.0	17.0	15.1	18.8	15.7	24.3	22.9
30 k–60 k	27.8	27.4	26.7	25.9	25.9	27.4	27.0	23.8	26.8
>60 k	22.3	19.9	30.3	36.0	39.2	34.9	40.3	25.2	27.5
Missing	24.1	23.5	23.0	21.2	19.7	19.0	17.1	26.8	22.8
Current marital status, %									
No	28.4	34.3	30.9	28.7	27.4	29.3	28.7	38.1	31.6
Yes	55.8	49.5	53.7	58.2	61.0	59.0	61.3	40.2	53.0
Missing	15.9	16.2	15.4	13.1	11.6	11.7	10.0	21.7	15.3
Vigorous exercise, %									
5+ times/week	13.6	11.8	11.6	13.7	15.0	14.9	16.6	11.3	12.4
2–4 times/week	23.9	21.7	26.8	32.1	33.1	29.3	31.6	20.4	25.7
1–4 times/month	20.2	20.6	23.8	23.0	22.9	23.0	22.3	21.1	22.2
Never/rarely	26.7	30.0	23.0	18.9	18.1	22.0	20.1	26.2	24.9
Missing	15.6	15.9	14.8	12.4	10.9	10.9	9.5	21.1	14.8
Smoking status, %									
Never	56.0	21.9	30.2	26.5	25.2	20.5	21.4	16.7	27.6
Ever	28.2	50.6	44.6	46.8	49.3	50.6	51.5	42.2	46.0
Current	8.3	21.7	16.0	15.5	14.1	19.3	15.8	35.0	18.2
Missing	7.6	5.8	9.3	11.2	11.5	9.7	11.4	6.1	8.2
DASH ^3^ score	23.7 (5.4)	23.1 (5.2)	23.8 (5.1)	24.7 (5.0)	25.4 (4.9)	24.8 (4.9)	25.6 (4.7)	23.3 (4.9)	23.8 (5.2)
Statin user, %	58.6	62.4	53.2	49.9	48.8	52.0	49.8	53.2	55.9
Atrial Fibrillation	8.1	8.6	6.7	7.3	8.0	7.6	7.7	6.5	7.6
Diabetes	30.4	31.9	24.4	16.3	13.7	13.4	11.0	15.7	24.8
Hypertension	69.1	71.5	62.4	60.1	60.5	64.4	62.8	68.5	65.9
Hyperlipidemia	68.2	70.0	64.5	62.4	61.4	63.6	63.4	63.1	66.1
Cancer	19.1	20.1	16.4	16.6	17.6	17.9	17.5	15.9	18.0
Depression	29.2	38.3	28.8	22.3	20.2	21.8	19.1	43.9	31.1
COPD	6.0	10.9	6.3	5.4	4.9	7.1	4.7	12.6	7.9
Stroke	4.9	5.4	3.2	2.5	2.5	2.8	2.3	2.9	3.9
Myocardial infarction	5.7	6.7	4.6	3.7	3.2	3.8	3.3	3.9	5.1

^1^ Mean (SD) for continuous variables and percentage (%) for categorical indicators. ^2^ AUD: alcohol use disorder. ^3^ DASH: Dietary Approaches to Stop Hypertension.

**Table 2:** Incidence rate and hazard ratios (HR, 95% CI) for heart failure by alcohol consumption.

			Current Drinkers (Drinks/Day)						*p* for Linear Trend	*p* for Non-Linear Trend
	Never Drinkers	Former Drinkers	0.1–0.5	0.6–1	1.1–2	2.1–3	3.1–4	AUD &/or Heavy Drinkers		
Heart Failure Events	3093	15715	11098	3391	1738	1506	320	1559		
Crude Incidence Rate ^1^	16.2	18.9	12.8	11.5	11.3	12.5	12.2	15.0		
Crude HR (95% CI)	1.00 (ref)	1.17 (1.13, 1.22)	0.79 (0.76, 0.82)	0.71 (0.68, 0.74)	0.70 (0.66, 0.74)	0.77 (0.73, 0.82)	0.75 (0.67, 0.84)	0.93 (0.88, 0.99)	<0.0001	<0.0001
Adjusted HR ^1^ (95% CI) ^2^	1.00 (ref)	1.21 (1.16, 1.26)	0.93 (0.89, 0.97)	0.80 (0.76, 0.84)	0.75 (0.70, 0.79)	0.83 (0.78, 0.88)	0.82 (0.73, 0.92)	1.10 (1.04, 1.17)	<0.0001	<0.0001
Adjusted HR ^2^ (95% CI) ^3^	1.00 (ref)	1.02 (0.98, 1.06)	0.90 (0.86, 0.94)	0.88 (0.84, 0.93)	0.86 (0.81, 0.91)	0.92 (0.86, 0.98)	0.95 (0.84, 1.06)	1.08 (1.01, 1.15)	0.53	<0.0001

^1^ Per 1000 person-years. ^2^ Adjusted HR 1: adjusted for age, gender, race/ethnicity, education level, marital status, and income level. ^3^ Adjusted HR 2: Further adjusted for exercise frequency, body mass index (kg/m^2^: <18.5, 18.5–22.4, 22.5–24.9, 25.0–27.4, 27.5–29.9, 30–32.4, 32.5–34.9, and ≥35), DASH score (quintiles), smoking, statin use, and baseline comorbidities including atrial fibrillation, diabetes, hypertension, hypercholesterolemia, depression, COPD, cancers, stroke, and myocardial infarction (all categories are the same as listed in Table 1 except BMI and DASH score). AUD: alcohol use disorder.

**Table 3:** Incidence rate and hazard ratios (HR, 95% CI) for HFrEF ^1^ by alcohol consumption.

			Current Drinkers (Drinks/Day)						*p* for Linear Trend	*p* for Non-Linear Trend
	Never Drinkers	Former Drinkers	0.1–0.5	0.6–1	1.1–2	2.1–3	3.1–4	AUD &/or Heavy Drinkers		
Heart Failure Events	1193	6198	4441	1400	722	614	132	656		
Crude Incidence Rate ^2^	6.5	7.9	5.3	4.9	4.8	5.3	5.2	6.5		
Crude HR (95% CI)	1.00 (ref)	1.20 (1.13, 1.28)	0.81 (0.76, 0.87)	0.75 (0.70, 0.81)	0.74 (0.68, 0.81)	0.81 (0.74, 0.90)	0.80 (0.67, 0.96)	1.01 (0.91, 1.11)	<0.0001	<0.0001
Adjusted HR ^1^ (95% CI) ^3^	1.00 (ref)	1.23 (1.15, 1.31)	0.95 (0.90, 1.02)	0.84 (0.78, 0.91)	0.80 (0.73, 0.87)	0.87 (0.79, 0.96)	0.87 (0.73, 1.04)	1.16 (1.06, 1.28)	<0.0001	<0.0001
Adjusted HR ^2^ (95% CI) ^4^	1.00 (ref)	1.04 (0.97, 1.10)	0.93 (0.87, 0.99)	0.93 (0.86, 1.00)	0.91 (0.83, 1.00)	0.95 (0.86, 1.05)	1.00 (0.84, 1.2)	1.13 (1.02, 1.24)	0.19	<0.0001

^1^ HFrEF: heart failure with reduced ejection fraction (i.e., ejection fraction is 40% or less) ^2^ Per 1000 person-years. ^3^ Adjusted HR 1: adjusted for age, gender, race/ethnicity, education level, marital status, and income level. ^4^ Adjusted HR 2: Further adjusted for exercise frequency, body mass index (kg/m^2^: <18.5, 18.5–22.4, 22.5–24.9, 25.0–27.4, 27.5–29.9, 30–32.4, 32.5–34.9, and ≥35), DASH score (quintiles), smoking, statin use, and baseline comorbidities including atrial fibrillation, diabetes, hypertension, hypercholesterolemia, depression, COPD, cancers, stroke, and myocardial infarction (all categories are the same as listed in Table 1 except BMI and DASH score). AUD: alcohol use disorder.

**Table 4:** Incidence rate and hazard ratios (HR, 95% CI) for HFpEF ^1^ by alcohol consumption.

			Current Drinkers (Drinks/Day)						*p* for Linear Trend	*p* for Non-Linear Trend
	Never Drinkers	Former Drinkers	0.1–0.5	0.6–1	1.1–2	2.1–3	3.1–4	AUD &/or Heavy Drinkers		
Heart Failure Events	1900	9517	6657	1991	1016	892	188	903		
Crude Incidence Rate ^2^	10.2	11.8	7.8	6.9	6.8	7.6	7.3	8.9		
Crude HR (95% CI)	1.00 (ref)	1.16 (1.10, 1.22)	0.77 (0.73, 0.81)	0.67 (0.63, 0.72)	0.66 (0.61, 0.71)	0.74 (0.68, 0.80)	0.72 (0.62, 0.83)	0.88 (0.81, 0.95)	<0.0001	<0.0001
Adjusted HR ^1^ (95% CI) ^3^	1.00 (ref)	1.21 (1.15, 1.27)	0.91 (0.86, 0.96)	0.76 (0.72, 0.81)	0.71 (0.66, 0.77)	0.80 (0.74, 0.87)	0.79 (0.68, 0.91)	1.06 (0.97, 1.14)	<0.0001	<0.0001
Adjusted HR ^2^ (95% CI) ^4^	1.00 (ref)	1.01 (0.96, 1.06)	0.88 (0.83, 0.93)	0.85 (0.80, 0.91)	0.83 (0.77, 0.90)	0.90 (0.83, 0.98)	0.92 (0.79, 1.07)	1.05 (0.96, 1.13)	0.74	<0.0001

^1^ HFpEF: heart failure with preserved ejection fraction (i.e., ejection fraction is 40% or less) ^2^ Per 1000 person years. ^3^ Adjusted HR 1: adjusted for age, gender, race/ethnicity, education level, marital status, and income level. ^4^ Adjusted HR 2: Further adjusted for exercise frequency, body mass index (kg/m^2^: <18.5, 18.5–22.4, 22.5–24.9, 25.0–27.4, 27.5–29.9, 30–32.4, 32.5–34.9, and ≥35), DASH score (quintiles), smoking, statin use, and baseline comorbidities including atrial fibrillation, diabetes, hypertension, hypercholesterolemia, depression, COPD, cancers, stroke and myocardial infarction (all categories are the same as listed in Table 1 except BMI and DASH score). AUD: alcohol use disorder.

**Table 5:** Incidence rate and hazard ratios (95% CI) for heart failure by beverage preference (269,518) ^1^.

Beverage Preference	Heart Failure Events	Crude Hazard Ratio and 95% CI	Adjusted Hazard Ratio ^2^ and 95% CI	Adjusted Hazard Ratio ^3^ and 95% CI	Adjusted Hazard Ratio ^4^ and 95% CI
Never drinkers	3093	1.00 (ref)	1.00 (ref)	1.00 (ref)	1.00 (ref)
No preference	6440	0.75 (0.72, 0.78)	0.75 (0.72, 0.79)	0.71 (0.68, 0.75)	0.93 (0.89, 0.98)
Preferred beer	6429	0.92 (0.88, 0.96)	0.88 (0.84, 0.92)	0.78 (0.74, 0.82)	1.02 (0.97, 1.07)
Preferred wine	3313	0.91 (0.87, 0.95)	0.91 (0.87, 0.95)	0.87 (0.83, 0.92)	0.97 (0.92, 1.02)
Preferred liquor	3430	0.9 (0.86, 0.94)	0.88 (0.84, 0.92)	0.86 (0.81, 0.9)	0.94 (0.89, 0.99)

^1^ In this table, we have excluded those in our study population who were former drinkers. ^2^ Adjusted HR 1: adjusted for age, gender, race/ethnicity, education level, marital status, and income level. ^3^ Adjusted HR 2: Further adjusted for exercise frequency, body mass index (kg/m^2^: <18.5, 18.5–22.4, 22.5–24.9, 25.0–27.4, 27.5–29.9, 30–32.4, 32.5–34.9, and ≥35), DASH score (quintiles), smoking, statin use, and baseline comorbidities including atrial fibrillation, diabetes, hypertension, hypercholesterolemia, depression, COPD, cancers, stroke, and myocardial infarction (all categories are the same as listed in Table 1 except BMI and DASH score). ^4^ Further adjusted to ethanol intake.

## Data Availability

Data described in the article, codebook, and analytic code will not be made available to other researchers for purposes of reproducing the results or replicating the procedure in order to comply with current VA privacy regulations pursuant to the US Department of Veterans Affairs policies on compliance with the confidentiality of US veterans’ data.

## Supplementary Materials

The following supporting information can be downloaded at: https://www.mdpi.com/article/10.3390/nu18030471/s1. Figure S1: Participant flow chart; Table S1: Comparison of baseline characteristics between included and excluded participants; Table S2: Comorbidities Diagnosis Codes; Table S3: Sensitivity analysis of the association between alcohol and incident heart failure; Table S4: Stratified analysis of the association between alcohol and incident heart failure; Table S5: Incidence rate and hazard ratios (95% CI) for heart failure by beverage preference (253,692) applying a preference cutoff of 70% ^1^; File S1: MVP Core Publication Acknowledgement_June 2025.

## References

[1] Roger V.L. (2021). Epidemiology of Heart Failure. Circ. Res..

[2] Heidenreich P.A., Bozkurt B., Aguilar D., Allen L.A., Byun J.J., Colvin M.M., Deswal A., Drazner M.H., Dunlay S.M., Evers L.R. (2022). 2022 AHA/ACC/HFSA Guideline for the Management of Heart Failure: A Report of the American College of Cardiology/American Heart Association Joint Committee on Clinical Practice Guidelines. Circulation.

[3] Domínguez F., Adler E., García-Pavía P. (2024). Alcoholic cardiomyopathy: An update. Eur. Heart J..

[4] Mukhopadhyay P., Yokus B., Paes-Leme B., Batkai S., Ungvári Z., Haskó G., Pacher P. (2025). Chronic alcohol consumption accelerates cardiovascular aging and decreases cardiovascular reserve capacity. GeroScience.

[5] Steiner J.L., Lang C.H. (2017). Etiology of alcoholic cardiomyopathy: Mitochondria, oxidative stress and apoptosis. Int. J. Biochem. Cell Biol..

[6] Griswold M.G., Fullman N., Hawley C., Arian N., Zimsen S.R.M., Tymeson H.D., Venkateswaran V., Tapp A.D., Forouzanfar M.H., Salama J.S. (2018). Alcohol use and burden for 195 countries and territories, 1990–2016: A systematic analysis for the Global Burden of Disease Study 2016. Lancet.

[7] CDC Alcohol Use and Your Health. https://www.cdc.gov/alcohol/about-alcohol-use/index.html.

[8] Gonçalves A., Claggett B., Jhund P.S., Rosamond W., Deswal A., Aguilar D., Shah A.M., Cheng S., Solomon S.D. (2015). Alcohol consumption and risk of heart failure: The Atherosclerosis Risk in Communities Study. Eur. Heart J..

[9] Walsh C.R., Larson M.G., Evans J.C., Djousse L., Ellison R.C., Vasan R.S., Levy D. (2002). Alcohol consumption and risk for congestive heart failure in the Framingham Heart Study. Ann. Intern. Med..

[10] Wood A.M., Kaptoge S., Butterworth A.S., Willeit P., Warnakula S., Bolton T., Paige E., Paul D.S., Sweeting M., Burgess S. (2018). Risk thresholds for alcohol consumption: Combined analysis of individual-participant data for 599 912 current drinkers in 83 prospective studies. Lancet.

[11] Djoussé L., Gaziano J.M. (2008). Alcohol consumption and heart failure: A systematic review. Curr. Atheroscler. Rep..

[12] Cosmi F., Di Giulio P., Masson S., Finzi A., Marfisi R.M., Cosmi D., Scarano M., Tognoni G., Maggioni A.P., Porcu M. (2015). Regular wine consumption in chronic heart failure: Impact on outcomes, quality of life, and circulating biomarkers. Circ. Heart Fail..

[13] Wollin S.D., Jones P.J.H. (2001). Alcohol, Red Wine and Cardiovascular Disease. J. Nutr..

[14] Joseph J., Liu C., Hui Q., Aragam K., Wang Z., Charest B., Huffman J.E., Keaton J.M., Edwards T.L., Demissie S. (2022). Genetic architecture of heart failure with preserved versus reduced ejection fraction. Nat. Commun..

[15] Patel Y.R., Robbins J.M., Kurgansky K.E., Imran T., Orkaby A.R., McLean R.R., Ho Y.-L., Cho K., Michael Gaziano J., Djousse L. (2018). Development and validation of a heart failure with preserved ejection fraction cohort using electronic medical records. BMC Cardiovasc. Disord..

[16] Gaziano L., Cho K., Djousse L., Schubert P., Galloway A., Ho Y., Kurgansky K., Gagnon D.R., Russo J.P., Di Angelantonio E. (2021). Risk factors and prediction models for incident heart failure with reduced and preserved ejection fraction. ESC Heart Fail..

[17] US Department of Health and Human Services Alcohol and Cancer Risk. https://www.hhs.gov/surgeongeneral/reports-and-publications/alcohol-cancer/index.html.

[18] Lutski M., Stark A.H., Dichtiar R., Lubel S.Y., Ornan E.M., Sinai T. (2025). Nutrition Facts Label Use and Adherence to the DASH Dietary Pattern: Results from a National Health and Nutrition Survey. Prev. Chronic Dis..

[19] Djoussé L., Ho Y., Nguyen X.T., Gagnon D.R., Wilson P.W.F., Cho K., Gaziano J.M., Halasz I., Federman D., Beckham J. (2018). DASH Score and Subsequent Risk of Coronary Artery Disease: The Findings From Million Veteran Program. J. Am. Heart Assoc..

[20] Piano M.R., Marcus G.M., Aycock D.M., Buckman J., Hwang C.-L., Larsson S.C., Mukamal K.J., Roerecke M. (2025). Alcohol Use and Cardiovascular Disease: A Scientific Statement From the American Heart Association. Circulation.

[21] Corporate Data Warehouse (CDW). https://www.hsrd.research.va.gov/for_researchers/cdw.cfm.

[22] Data Standardization—OHDSI. https://www.ohdsi.org/data-standardization/.

[23] Yeo Y., Jeong S.-M., Shin D.W., Han K., Yoo J., Yoo J.E., Lee S.-P. (2022). Changes in Alcohol Consumption and Risk of Heart Failure: A Nationwide Population-Based Study in Korea. Int. J. Environ. Res. Public Health.

[24] Arafa A., Kashima R., Kokubo Y., Teramoto M., Sakai Y., Nosaka S., Kawachi H., Shimamoto K., Matsumoto C., Gao Q. (2023). Alcohol consumption and the risk of heart failure: The Suita Study and meta-analysis of prospective cohort studies. Environ. Health Prev. Med..

[25] Larsson S.C., Wallin A., Wolk A. (2018). Alcohol consumption and risk of heart failure: Meta-analysis of 13 prospective studies. Clin. Nutr..

[26] Yousaf H., Rodeheffer R.J., Paterick T.E., Ashary Z., Ahmad M.N., Ammar K.A. (2014). Association between alcohol consumption and systolic ventricular function: A population-based study. Am. Heart J..

[27] Ho J.E., Enserro D., Brouwers F.P., Kizer J.R., Shah S.J., Psaty B.M., Bartz T.M., Santhanakrishnan R., Lee D.S., Chan C. (2016). Predicting Heart Failure With Preserved and Reduced Ejection Fraction: The International Collaboration on Heart Failure Subtypes. Circ. Heart Fail..

[28] Rasoul D., Ajay A., Abdullah A., Mathew J., Lee Wei En B., Mashida K., Sankaranarayanan R. (2023). Alcohol and Heart Failure. Eur. Cardiol..

[29] Wong B., Moore A., McDonald K., Ledwidge M. (2024). Alcohol Consumption and Progression of Heart Failure in Those at Risk for or with Pre-heart Failure. J. Card. Fail..

[30] Abramson J.L., Williams S.A., Krumholz H.M., Vaccarino V. (2001). Moderate Alcohol Consumption and Risk of Heart Failure Among Older Persons. JAMA.

[31] Fernández-Solà J. (2015). Cardiovascular risks and benefits of moderate and heavy alcohol consumption. Nat. Rev. Cardiol..

[32] Godos J., Caraci F., Micek A., Castellano S., D’Amico E., Paladino N., Ferri R., Galvano F., Grosso G. (2021). Dietary Phenolic Acids and Their Major Food Sources Are Associated with Cognitive Status in Older Italian Adults. Antioxidants.

[33] Humans IWG on the E of CR to (1988). Alcohol Drinking.

[34] Castaldo L., Narváez A., Izzo L., Graziani G., Gaspari A., Minno G.D., Ritieni A. (2019). Red Wine Consumption and Cardiovascular Health. Molecules.

[35] Klatsky A.L., Chartier D., Udaltsova N., Gronningen S., Brar S., Friedman G.D., Lundstrom R.J. (2005). Alcohol drinking and risk of hospitalization for heart failure with and without associated coronary artery disease. Am. J. Cardiol..

