# Supplementary material for: Alcohol Intake and Incidence of Heart Failure and Its Subtypes: VA Million Veteran Program
Source: Nutrients. 2026 Jan 31;18(3):471. doi: 10.3390/nu18030471 (PMC12899092; doi:10.3390/nu18030471)
Supplement: Supplementary file 1 [file nutrients-18-00471-s001.zip › nutrients-4104322-supplementary/Supplement materials.pdf]

Supplemental Figure S1. Participant flow chart.

1,016,584 veterans enrolled VA Million Veteran Program (MVP) as of September 2024

↓

475,121 participants who completed the MVP Lifestyle Surveys

- excluded 37,574 participants with heart failure prior to or at baseline
- excluded 662 implausible death records
- excluded 19,168 non-records of clinical visit after baseline
- excluded 16,369 participants who did not respond to FFQ or with missing data on alcohol intakes

↓

study population: 401,348 participants

Supplemental Table S1. Comparison of baseline characteristics between included and excluded participants

Characteristic	**Included in the Study**	**Excluded from the study**
N	401,348	73,773
Age, year	65.2 (12.4)	69.4 (11.1)
Body Mass Index, kg/m^2	29.3 (5.4)	29.9 (6.1)
Female, %	9.4	5.8
Race and ethnicity, %		
Non-Hispanic White	79.4	80.4
Non-Hispanic Black	10.5	10.8
Hispanic	6.6	5.5
Others	3.4	3.3
Education level, %		
High school	2.2	3.7
Some college	16.5	17.7
College or above	66.6	62.5
Missing	14.8	16.1
Family annual income, %		
<30k	22.9	24.2
30k-60k	26.8	23.4
>60k	27.5	28.3
Missing	22.8	24.1
Current marital status, %		
No	31.6	31.1
Yes	53.0	52.5
Missing	15.3	16.4
Vigorous exercise, %		
5+ times/week	12.4	11.6
2-4 times/week	25.7	22.5
1-4 times/month	22.2	19.5
Never/rarely	24.9	30.1
Missing	14.8	16.3
Smoking status, %		
Never	27.6	18.8
Ever	46.0	43.5
Current	18.2	15.9
Missing	8.2	21.8
DASH score	23.8 (5.2)	23.7 (5.1)
Statin user, %	55.9	61.3
Baseline comorbidities, %		
Atrial Fibrillation	7.6	24.1
Diabetes	24.8	32.1
Hypertension	65.9	67.8
Hyperlipidemia	66.1	63.6
Cancer	18.0	19.2
Depression	31.1	28.4
COPD	7.9	16.5
Stroke	3.9	7.9
Myocardial infarction	5.1	5.1

[1]Mean (SD) for continuous variables and percentage (%) for categorical indicators; DASH: Dietary Approaches to Stop Hypertension

Supplementary Table S2. Comorbidities Diagnosis Codes

Disease	ICD Diagnostic Codes used to Identify Cases
Hypertension	ICD9: 401*[1], 402*, 403*, 404*, 405*, 437.2*
	ICD10: I10*, I11*, I12*, I13*, I15*, I67.4*
Hyperlipidemia	ICD9: 272.4*, 272.2*
	ICD10: E78.2*, E78.4*, E78.5*
Atrial Fibrillation	ICD9: 427.3*
	ICD10: I48*
Diabetes	ICD9: 250*
	ICD10: E08*, E09*, E10*, E11*, E13*
All Cancers[2]	ICD9: 14*, 15*, 16*, 170*, 171*, 172*, 174*, 175*, 176*, 177*, 178*, 179*,18*, 19*, 20*
	ICD10: C*, except C44*, D01.[012]*, D02.[012]*, D03.[012]*, D05*, D07.[05]*, D3A*, D41.[012]*, D45.*, M84.45[1239]A*, M84.55[01239]A*, M84.65[01239]A*, M84.48XA, M84.58XA, M84.68XA
COPD	ICD9: 115, 115.01, 115.02, 115.03, 115.04, 115.05, 115.09, 115.1, 115.11, 115.12, 115.13, 115.14, 115.15, 115.19, 115.9, 115.91, 115.92, 115.93, 115.94, 115.95, 115.99, 490, 491.1, 491.2, 491.21, 491.21, 491.21, 491.2, 491.2, 491.8, 491.8, 494.1, 496, 748.61, V81.3
	ICD10: J40, J41.0, J41.1, J41.8, J42., J43.0, J43.1, J43.2, J43.8, J43.9, J44.0, J44.1, J44.9, J47.0, J47.1, J47.9
Depression	ICD9: 296.20, 296.21, 296.22, 296.23, 296.24, 296.25, 296.26, 296.30, 296.31, 296.32, 296.33, 296.34, 296.35, 296.36, 300.4, 311
	ICD10: F32.0, F32.1, F32.2, F32.3, F32.4, F32.5, F32.9, F33.0, F33.1, F33.2, F33.3, F33.40, F33.41, F33.42, F33.9, F34.1
Stroke	ICD 9: 430.x, 431.x, 436.x, 433.01, 433.1, 433.11, 433.21, 433.31, 433.81, 433.91, 434.0, 434.00, 434.01,434.1, 434.10, 434.11, 434.9, 434.90, 434.91, 436., 437.0, 437.6
	ICD10: I60.x, I61.x, I63.019, I63.039, I63.119, I63.139, I63.20, I63.219, I63.22, I63.239, I63.30, I63.319, I63.329, I63.339, I63.349, I63.40, I63.419, I63.429, I63.439, I63.449, I63.50, I63.519, I63.529, I63.539, I63.549, I63.59, I67.89
Myocardial infarction	410.x, 411.x, 412.x
	I21.x, I22.x, I25.2, I23.x

[1]*: wildcard

Supplemental Table S3. Sensitivity analysis of the association between alcohol and incident heart failure

	Never drinkers	Former drinkers	Current Drinkers (drinks/day)					
			0.1-0.5	0.6-1	1.1-2	2.1-3	3.1-4	AUD &/or heavy drinkers
Excluded <2 years of follow-up time	1.00 (ref)	1.04 (0.99, 1.09)	0.93 (0.89, 0.98)	0.91 (0.86, 0.96)	0.91 (0.85, 0.97)	0.97 (0.90, 1.04)	0.98 (0.86, 1.12)	1.14 (1.06, 1.23)
Excluded participants with missing covariates	1.00 (ref)	1.02 (0.97, 1.07)	0.90 (0.86, 0.95)	0.89 (0.84, 0.94)	0.86 (0.81, 0.93)	0.93 (0.86, 1.00)	0.96 (0.84, 1.09)	1.10 (1.02, 1.18)
Imputed missing values of covariates	1.00 (ref)	1.02 (0.98, 1.06)	0.90 (0.86, 0.93)	0.87 (0.83, 0.92)	0.85 (0.80, 0.90)	0.91 (0.86, 0.97)	0.93 (0.83, 1.05)	1.09 (1.02, 1.16)
Included death from heart failure as cases	1.00 (ref)	1.01 (0.97, 1.05)	0.90 (0.86, 0.93)	0.88 (0.84, 0.92)	0.86 (0.81, 0.92)	0.92 (0.86, 0.97)	0.92 (0.82, 1.04)	1.08 (1.01, 1.14)
Competing risk model of HF and mortality	1.00 (ref)	1.10 (1.07, 1.13)	0.93 (0.90, 0.95)	0.80 (0.77, 0.84)	0.74 (0.69, 0.77)	0.82 (0.78, 0.87)	0.84 (0.75, 0.93)	1.10 (1.04, 1.16)
Excluded patients with CVD at baseline	1.00 (ref)	1.02 (0.98, 1.07)	0.90 (0.86, 0.94)	0.88 (0.83, 0.93)	0.86 (0.80, 0.92)	0.95 (0.88, 1.01)	0.94 (0.83, 1.06)	1.09 (1.02, 1.17)

[1]Per 1000 person years.

[2]Adjusted HR 1: adjusted for age, gender, race/ethnicity, education level, marital status, and income level.

[3]Adjusted HR 2: Further adjusted for exercise frequency, body mass index (kg/m^2: <18.5, 18.5-22.4, 22.5-24.9, 25.0-27.4, 27.5-29.9, 30-32.4, 32.5-34.9, and ≥35), DASH score (quintiles), smoking, statin use, and baseline comorbidities including atrial fibrillation, diabetes, hypertension, hypercholesterolemia, depression, COPD, cancers, stroke and myocardial infarction (all categories are the same as listed in Table 1 except BMI and DASH score).

AUD: alcohol use disorder; CVD: cardiovascular diseases included stroke and myocardial infarction.

Supplemental Table S4. Stratified analysis of the association between alcohol and incident heart failure

	Never drinkers	Former drinkers	Current Drinkers (drinks/day)					
			0.1-0.5	0.6-1	1.1-2	2.1-3	3.1-4	AUD &/or heavy drinkers
Male	1.00 (ref)	1.01 (0.97, 1.06)	0.90 (0.86, 0.94)	0.88 (0.84, 0.93)	0.86 (0.81, 0.92)	0.92 (0.87, 0.98)	0.95 (0.85, 1.07)	1.08 (1.02, 1.15)
Female	1.00 (ref)	1.05 (0.89, 1.22)	0.89 (0.76, 1.05)	0.89 (0.69, 1.15)	0.83 (0.56, 1.23)	0.75 (0.48, 1.15)	0.54 (0.13, 2.16)	0.83 (0.54, 1.27)
Age < 65 years	1.00 (ref)	1.01 (0.94, 1.10)	0.88 (0.81, 0.95)	0.83 (0.75, 0.92)	0.80 (0.70, 0.91)	0.91 (0.80, 1.03)	0.94 (0.73, 1.20)	1.08 (0.97, 1.20)
Age ≥65 years	1.00 (ref)	1.01 (0.96, 1.05)	0.91 (0.86, 0.95)	0.89 (0.84, 0.94)	0.87 (0.82, 0.94)	0.91 (0.85, 0.98)	0.93 (0.82, 1.06)	1.06 (0.98, 1.14)
Never smoking	1.00 (ref)	1.00 (0.94, 1.07)	0.89 (0.84, 0.95)	0.86 (0.78, 0.94)	0.83 (0.74, 0.93)	0.89 (0.78, 1.02)	0.82 (0.61, 1.10)	1.01 (0.87, 1.18)
Ever smoking	1.00 (ref)	1.05 (0.99, 1.11)	0.93 (0.88, 0.98)	0.91 (0.85, 0.97)	0.90 (0.83, 0.97)	0.96 (0.88, 1.03)	1.00 (0.88, 1.14)	1.13 (1.05, 1.22)
Not or less exercise	1.00 (ref)	1.02 (0.95, 1.09)	0.89 (0.83, 0.95)	0.86 (0.79, 0.93)	0.88 (0.80, 0.97)	0.89 (0.80, 0.99)	0.86 (0.71, 1.04)	1.12 (1.00, 1.26)
Regular exercise (>1/w)	1.00 (ref)	1.02 (0.97, 1.07)	0.90 (0.86, 0.96)	0.90 (0.84, 0.97)	0.83 (0.76, 0.91)	0.94 (0.86, 1.02)	0.99 (0.84, 1.16)	1.08 (0.99, 1.17)

[1]adjusted for age, gender, race/ethnicity, education level, marital status, income level, exercise frequency, body mass index (kg/m^2: <18.5, 18.5-22.4, 22.5-24.9, 25.0-27.4, 27.5-29.9, 30-32.4, 32.5-34.9, and ≥35), DASH score (quintiles), smoking, statin use, and baseline comorbidities including atrial fibrillation, diabetes, hypertension, hypercholesterolemia, depression, COPD, cancers, stroke and myocardial infarction, except the stratified factor.

Supplemental Table S5. Incidence rate and hazard ratios (95% CI) for heart failure by beverage preference (253,692) applying a preference cutoff of 70%[1]

Beverage preference	Heart Failure Events	Crude Hazard Ratio and 95% CI	Adjusted Hazard Ratio[2] and 95% CI	Adjusted Hazard Ratio[3] and 95% CI	Adjusted Hazard Ratio[4] and 95% CI
Never drinkers	3093	1.00 (ref)	1.00 (ref)	1.00 (ref)	1.00 (ref)
No preference	8464	0.73 (0.70, 0.76)	0.78 (0.75, 0.82)	0.73 (0.69, 0.77)	0.97 (0.92, 1.02)
Preferred beer	5592	0.88 (0.85, 0.92)	0.90 (0.87, 0.95)	0.79 (0.75, 0.83)	1.05 (1.00, 1.11)
Preferred wine	2739	0.90 (0.86, 0.93)	0.92 (0.88, 0.96)	0.88 (0.83, 0.93)	0.98 (0.93, 1.04)
Preferred Liquor	2817	0.88 (0.84, 0.92)	0.90 (0.85, 0.94)	0.86 (0.82, 0.91)	0.96 (0.91, 1.01)

[1]In this table, we have excluded those in our study population who were former drinkers

[2]Adjusted HR 1: adjusted for age, gender, race/ethnicity, education level, marital status, and income level.

[3]Adjusted HR 2: Further adjusted for exercise frequency, body mass index (kg/m^2: <18.5, 18.5-22.4, 22.5-24.9, 25.0-27.4, 27.5-29.9, 30-32.4, 32.5-34.9, and ≥35), DASH score (quintiles), smoking, statin use, and baseline comorbidities including atrial fibrillation, diabetes, hypertension, hypercholesterolemia, depression, COPD, cancers, stroke and myocardial infarction (all categories are the same as listed in Table 1 except BMI and DASH score).

[4]Further adjusted to ethanol intake.
